# Patients' experiences of primary healthcare and dermatology provision for alopecia

**DOI:** 10.1002/ski2.324

**Published:** 2023-12-10

**Authors:** Fabio Zucchelli, Marije van Dalen, Nick Sharratt, Amy Johnson, Jen Chambers

**Affiliations:** ^1^ The Centre for Appearance Research University of the West of England Bristol UK; ^2^ Department of Child and Adolescent Psychiatry/Psychology Erasmus MC Sophia Children's Hospital Rotterdam the Netherlands; ^3^ Alopecia Shipley UK

## Abstract

**Background:**

Alopecia describes a group of dermatological conditions characterised by hair loss, which are either non‐scarring or scarring in nature, and range from bald patches to complete body hair loss, to general thinning. In the UK, the General Practitioner (GP) is typically the first point of contact, and some patients are referred for specialist dermatology consultation. However, little is known about how individuals with alopecia in the UK experience the care provided by the National Health Service.

**Objectives:**

We aimed to understand patients' perceptions of primary healthcare and dermatology provision. Further, we aimed to investigate how care provision and patients' overall patient journey might be improved in the UK, and how these lessons may apply internationally.

**Methods:**

An online mixed methods survey was distributed by Alopecia UK to UK‐based individuals with alopecia. Open‐ended text responses were analysed using qualitative content analysis. Quantitative data were analysed using descriptive analyses and dependent measures *t*‐tests.

**Results:**

A total of 291 participants completed the survey. They reported neutral‐to‐partial dissatisfaction with their GP appointments, with greater satisfaction in their most recent compared to their first appointment. Participants highlighted positive experiences with GPs and dermatologists as well as areas for improvement. Participants also expressed a desire for a greater degree of support and understanding about the psychological impact of alopecia.

**Conclusions:**

Results highlight the importance of being empathic and caring healthcare professionals for patients with alopecia, the need for training for GPs on alopecia, as well as a simplified and joined up pathway between primary and secondary healthcare.

## Graphical abstract

This study sought the subjective experiences of patients with alopecia in their healthcare consultations. Findings suggest health professionals' empathy for the psychosocial impact of alopecia is a crucial element in patients' experience.

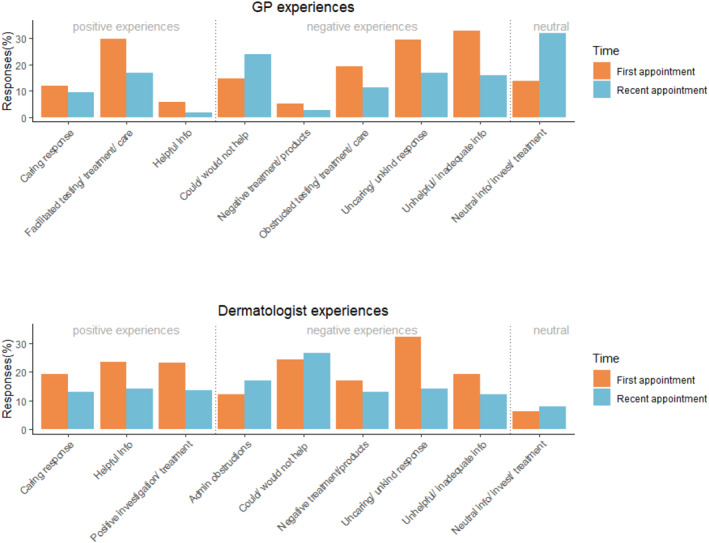

**What is already known about this topic?**

- In the UK, the General Practitioner (GP) is typically the first point of contact for patients. Patients may then be referred to a dermatologist. Guidelines exist for GPs and dermatologists to aid them in providing information and possible treatment. However, it is not yet known how patients experience their healthcare provision.

**What does this study add?**

- Patients reported being mildly dissatisfied to neutral experiences with their GP consultations and neutral experiences with the dermatologists' consultations. A third of patients reported their GP as lacking relevant knowledge. Patients also highlighted a need for a more joined up and simple care pathway between primary care, secondary care, mental health services and third‐party providers.

**What are the clinical implications of this work?**

- This work shows that an empathetic and caring response is especially valuable to patients. In addition, to help health care providers manage patients' expectations and optimise overall patient experience, it appears important to increase GPs' medical knowledge on alopecia, and for all health professionals including dermatologists to understand the potential psychosocial impact (positive or negative) of their consultations, and to employ empathic communication when consulting affected individuals.

## 1 INTRODUCTION

Alopecia refers to any form of hair loss, though the term is most commonly associated with Alopecia Areata (AA). Alopecia Areata is a dermatological autoimmune condition that causes hair loss ranging from patches on the scalp or body (patchy AA), to complete hair loss of the scalp (AA totalis), and hair loss on the entire body (AA universalis^1^). An AA cumulative lifetime incidence of around 2%–2.5% has been reported across Western countries, with peak onset at 25–29 years.^2^ Scarring alopecia, in which hair follicles are irreversibly destroyed leaving scar tissue at the site of hair loss, usually occurs in early‐to‐middle adulthood,^3^ or in the case of the Frontal Fibrosing Alopecia variant, in postmenopausal women.^4^ Androgenetic alopecia, more commonly known as pattern baldness, occurs in over half of men over 50 and in 40% of women over 60.^3^

Alopecia of all forms can have a profound impact on individuals' psychological well‐being, perhaps unsurprisingly given their chronic clinical course and their effects on appearance. Further stressors in AA include unpredictable prognosis and relapse, and in scarring alopecia, pain and itching.^3^ In patients with AA, international meta‐analyses show lower health‐related quality of life compared to matched controls,^5^, ^6^ and primary studies suggest an elevated incidence of anxiety (including social anxiety) and depression,^7^, ^8^, ^9^ which do not seem to be related to AA severity.^10^ Androgenetic alopecia is also associated with decreased quality of life, especially in women.^3^ Fewer data exist on the psychosocial effects of scarring alopecia, though a review of the three studies that have examined this issue in women points to a similarly negative impact.^3^

For individuals with AA, the GP situated in Primary Health Care is generally the first point of healthcare contact,^11^ though this may vary internationally depending on healthcare models. From recent data in the UK, 24% of people who visited their GP were subsequently referred for specialist dermatology review in the first year after receiving their diagnosis.^11^ Of those who visited their GP, 46% were not prescribed any medication,^11^ while in the Unites States one‐quarter of alopecia patients were prescribed treatment within a week of diagnosis, increasing to over half after 12 months.^12^ In the UK, practice guidelines indicate that AA patients should be selectively referred to a dermatologist,^13^ such as when a scalp biopsy is needed or the diagnosis is uncertain.

The UK National Institute for Health and Care Excellence (NICE) guidelines on AA and androgenetic alopecia^13^, ^14^ advise GPs to provide information about the condition and treatment options, and signpost available support services. Guidelines on AA issued by the British Association of Dermatologists^15^ include information on treatment and recommendations for referral to psychologists, but are limited on the information they contain on psychological impact and support. There are no formal guidelines for scarring alopecia and Primary Care Dermatology Network guidelines on Lichen Planus do not mention psychosocial impact or psychosocial support services at all.^16^ It remains unclear how patients experience National Health Service (NHS) care provision in primary care and dermatology services informed by these guidelines. There is some existing research on people's experiences of wig provision,^9^ but not of their overall patient journey.

The aim of this survey study is to examine patients' experiences of NHS care provision in the UK from primary care and Dermatology services. More specifically, we aim to understand 1) how the current NHS care provision is perceived by patients with alopecia and 2) how NHS care provision and the overall patient journey might be improved. Though focused on a UK context, we hope that addressing these questions will also provide useful direction for health care providers internationally, by offering insights into broadly applicable health care delivery principles.

## 2 MATERIALS AND METHODS

The study was initiated by Alopecia UK and analysed by independent academic researchers. Ethical approval was granted by the faculty ethics committee from the researcher's institution prior to analyses. The qualitative aspect of this paper was written in accordance with published standards for reporting qualitative research.^17^

### 2.1 Participants

Participants were patients with alopecia living in the UK. As the sample was designed to represent the wide range of characteristics supported by Alopecia UK, there were no exclusion criteria based on participants' alopecia type or age. Data collection took place between June and August 2019.

### 2.2 Materials/survey

An online survey was created by two staff members of Alopecia UK, a national charity working to improve the lives of those affected by alopecia through support, awareness and research. The survey addressed individuals' experiences of consulting health professionals for alopecia diagnosis and treatment, and NHS wig provision. This paper focuses on individuals' experiences of alopecia diagnosis and treatment (participants' experiences of wig provision has been published elsewhere^18^).

The survey consisted of a mix of multiple choice questions (e.g., “In which year did your first GP appointment regarding your hair loss take place?”), Likert‐type satisfaction scales from 1 (very dissatisfied) to 5 (very satisfied) (e.g., “Thinking about your FIRST GP appointment regarding your hair loss, how satisfied were you with the level of care and advice provided to you by your GP?”) and invitations to provide textual elaboration on their ratings (e.g., “Tell us more about your first GP experience and why you have selected your chosen answer.”).

### 2.3 Procedure

The survey, hosted on SurveyMonkey, was distributed by Alopecia UK through their social media, newsletters, email lists and website. After gaining ethical approval from the researchers' institution, one Alopecia UK member fully anonymised the dataset on MS Excel 365, including deidentifying any textual responses. They then shared the data with academic researchers for analysis.

### 2.4 Data analysis

Quantitative data were analysed using IBM SPSS statistics version 27.^19^ Descriptive statistics were calculated for multiple choice questions and Likert scales. Satisfaction ratings for NHS appointments were compared using paired sampled *t*‐tests. Cohen's d was calculated to measure effect size, with *d* = 0.20 considered a small effect, *d* = 0.50 a medium effect and *d* = 0.80 a large effect.^20^ Differences in satisfaction ratings were visualised using raincloud plots^21^ in RStudio.^22^

Qualitative data in the form of open‐text responses were analysed by two researchers using inductive content analysis.^23^ They adopted a pragmatic research paradigm, characterised by ontological and epistemological flexibility, with a defined focus on solving practical “real‐world” problems, suited to the study's goals of understanding patients' experiences to inform healthcare delivery.^24^ Both researchers were men in their 30's with no first‐hand experience of alopecia, and with experience in qualitative health and appearance research, including participants with alopecia.

The researchers first familiarised themselves with the full textual dataset in MS Excel 365 by reading all content. One researcher (FZ) focused on participants' responses pertaining to GP experiences and improvements, and developed initial codes representing patterns of meaning based on these data at their manifest level, and a second researcher (NS) did likewise for all dermatology‐focused data. The researchers then held a consensus meeting in which they shared and discussed their respective codes, and checked these against the corresponding data for fit. At this meeting they created codebooks for (a) GP experiences, (b) Dermatology experiences and (c) suggested improvements, by mutually adjusting and/or collapsing codes, abstracting codes into broader categories, agreeing on code definitions and coding procedures. Any discrepancies were resolved until a consensus was reached. The two researchers then independently coded 10% of all data using the codebook until an intercoder consistency of at least 80% was reached.^25^ Subsequently, both independently coded their allocated remaining data.

## 3 RESULTS

### 3.1 Sample characteristics

A total of 361 people started the survey and 291 people (80.61%) completed the survey. The majority were diagnosed with patchy AA or AA universalis. Most participants lived in England and almost everyone had seen a GP and dermatologist for their alopecia. Further sample characteristics are shown in Table 1.

**TABLE 1:** Sample characteristics.

Characteristic	Total (*N* = 361)
Type of alopecia, *n* (%)	
Alopecia areata	124 (34.44)
Alopecia areata universalis	122 (33.89)
Alopecia areata totalis	44 (12.22)
Androgenetic alopecia	21 (5.83)
Frontal fibrosing alopecia	33 (9.17)
Lichen planopilaris	11 (3.06)
Other	5 (1.39)
Country of residence, *n* (%)	
England	300 (83.10)
Scotland	43 (11.91)
Wales	11 (3.05)
Northern Ireland	7 (1.94)
Health professionals seen, *n* (%)	
GP	344 (95.29)
Dermatologist	319 (88.37)
Trichologist	58 (16.07)
Dermatology nurse	36 (9.97)
Endocrinologist	5 (1.39)
Alternative medicine professional	5 (1.39)
Other	14 (3.88)
Years since diagnosis, mean (SD)	12.43 (13.31)

No demographic data were collected for gender, age or ethnicity/race. However, estimates of gender made by the fourth author based on email addresses provided by participants suggested that, of those who provided email addresses, 210 participants (96.33%) were female. Alopecia has an average onset of 25–29 years,^11^ and the average time since diagnosis in our sample is 12.43 years, so the average age of our sample is likely to be 37 – 42.

### 3.2 Experiences with National Health Service GPs

Detailed information on when people consulted their GPs and their satisfaction ratings are shown in Table 2. Participants were more satisfied with their most recent appointment than their first appointment (*t*(219) = 3.06, *p* = 0.003, *d* = 0.21).

**TABLE 2:** Years and satisfaction rates for General Practitioner (GP) and dermatologist appointments.

	GP	Dermatologist
Year of first appointment, *n* (% from total item responses)		
Before 1995	52 (16.30)	48 (15.79)
1995–2004	41 (12.85)	29 (9.54)
2005–2014	99 (31.03)	96 (31.58)
2015–2017	83 (26.02)	79 (25.99)
2018	25 (7.84)	29 (9.54)
2019	19 (5.96)	23 (7.57)
Satisfaction with first appointment, *n* (% from total item responses)		
Very dissatisfied	90 (28.13)	54 (17.53)
Fairly dissatisfied	58 (18.13)	58 (18.83)
Neutral	70 (21.88)	63 (20.45)
Fairly satisfied	70 (21.88)	72 (23.38)
Very satisfied	32 (10.00)	61 (19.81)
Year of most recent appointment, *n* (% from total item responses)		
Before 1995	15 (6.00)	13 (5.49)
1995–2004	16 (6.40)	16 (6.75)
2005–2014	43 (17.20)	31 (13.08)
2015–2017	65 (26.00)	55 (23.21)
2018	44 (17.60)	33 (13.92)
2019	67 (26.80)	89 (37.55)
Satisfaction with most recent appointment, *n* (% from total item responses)		
Very dissatisfied	44 (19.91)	52 (23.74)
Fairly dissatisfied	29 (13.12)	33 (15.07)
Neutral	88 39.82)	45 (20.55)
Fairly satisfied	27 (12.22)	48 (21.92)
Very satisfied	33 (14.93)	41 (18.72)

**FIGURE 1:**
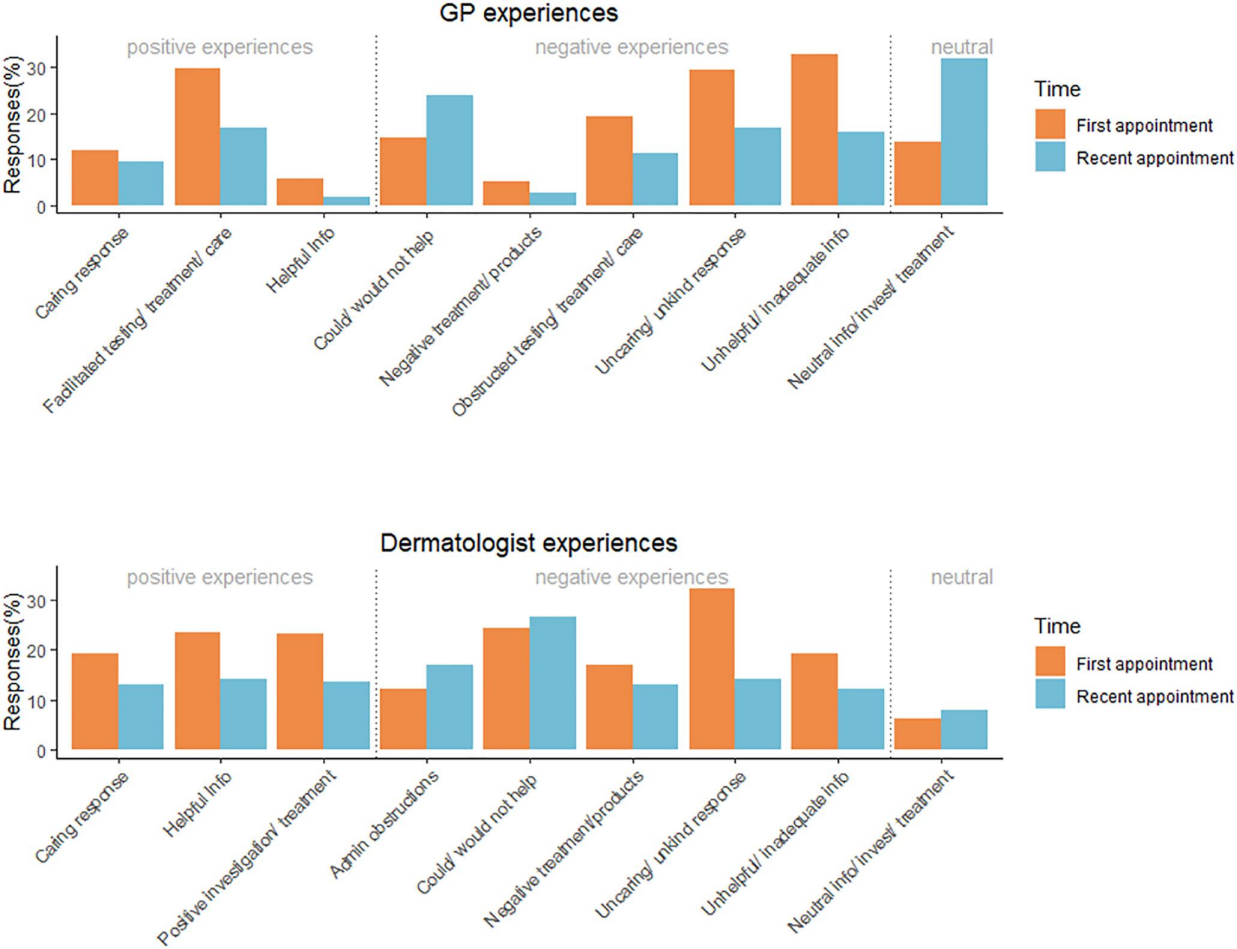
Coded responses for first and most recent General Practitioner (GP) and dermatologist experiences.

Content analysis results from the free‐text responses regarding GP experiences are shown in Figure 1, with code descriptions shown in Supplementary Table 1. The below text provides selected codes from the broader categories of positive and negative experiences, and example responses.

### 3.3 Positive experiences

About a third of participants felt that their GP facilitated testing, treatment or care, for example:

GP was very understanding of my concerns and outlined the ways forward. She referred me to a dermatologist. (From first GP experience)

Only a small number reported other positive experiences such as receiving helpful information or receiving a caring response, with especially low occurrences in participants' most recent appointment.

### 3.4 Negative experiences

Participants most commonly indicated having negative experiences in terms of being given unhelpful or inadequate information, and of receiving an unkind or uncaring response during their first GP experience, with around a third of participants reporting each:

They don’t know much about alopecia and blame stress (most recent GP experience).

GP told me to be grateful that it was only my hair I was losing and not a vital organ like liver or kidneys (first GP experience).

The rates of such negative experiences dropped by around a half in participants' responses concerning their most recent experience.

### 3.5 Experiences with dermatologists

From 361 participants who started the survey, 308 (85.3%) indicated they had seen a dermatologist. Detailed information on when people consulted their dermatologist and their satisfaction are shown in Table 2. There were no significant differences in participants' satisfaction ratings between the first and most recent appointment (*t*(218) = −1.96, *p* = 0.051, *d* = 0.13), though there was a trend towards greater satisfaction with first appointments.

Results from the content analysis of responses to dermatologist experience questions are shown in Figure 1. Codes were grouped into broader categories of positive, negative and neutral experiences.

### 3.6 Positive experiences

Between a fifth and a quarter of respondents gave responses coded into each of the codes described as receiving a caring response, helpful information, and a positive investigation and treatment experience in their first dermatologist appointment (though this reduced to under 15% in their most recent appointment):

Dermatologist immediately made diagnosis, stated no treatment has evidence of efficacy but was kind and gave me time to process as well as reassuring my little boy who was with me that it was a secret that “bald mummies are the coolest”‐ totally thoughtful, honest, empathetic and just wonderful.

Explained lots of things about alopecia that we didn’t know and answered all our questions.

They were very kind. Discussed different treatments and started me on one straight away.

### 3.7 Negative experiences

About a third of participants described receiving an unkind or uncaring response during their first dermatologist appointment:

I was young and self‐conscious. I felt bullied into letting student doctors come into my appointment where they proceeded to touch my head etc.

This rate approximately halved for participants' most recent dermatologist appointment. Around a fifth also reported receiving unhelpful information in their first appointment:

Nothing was really explained, just told it's AA and will probably grow back in time.

A quarter of participants reported that their dermatologist could not or would not help treat or manage their alopecia, in both their first and most recent appointments:

She said my hair would never grow back and there was nothing she could do.

### 3.8 Comparison between General Practitioner and Dermatologist consultations

**FIGURE 2:**
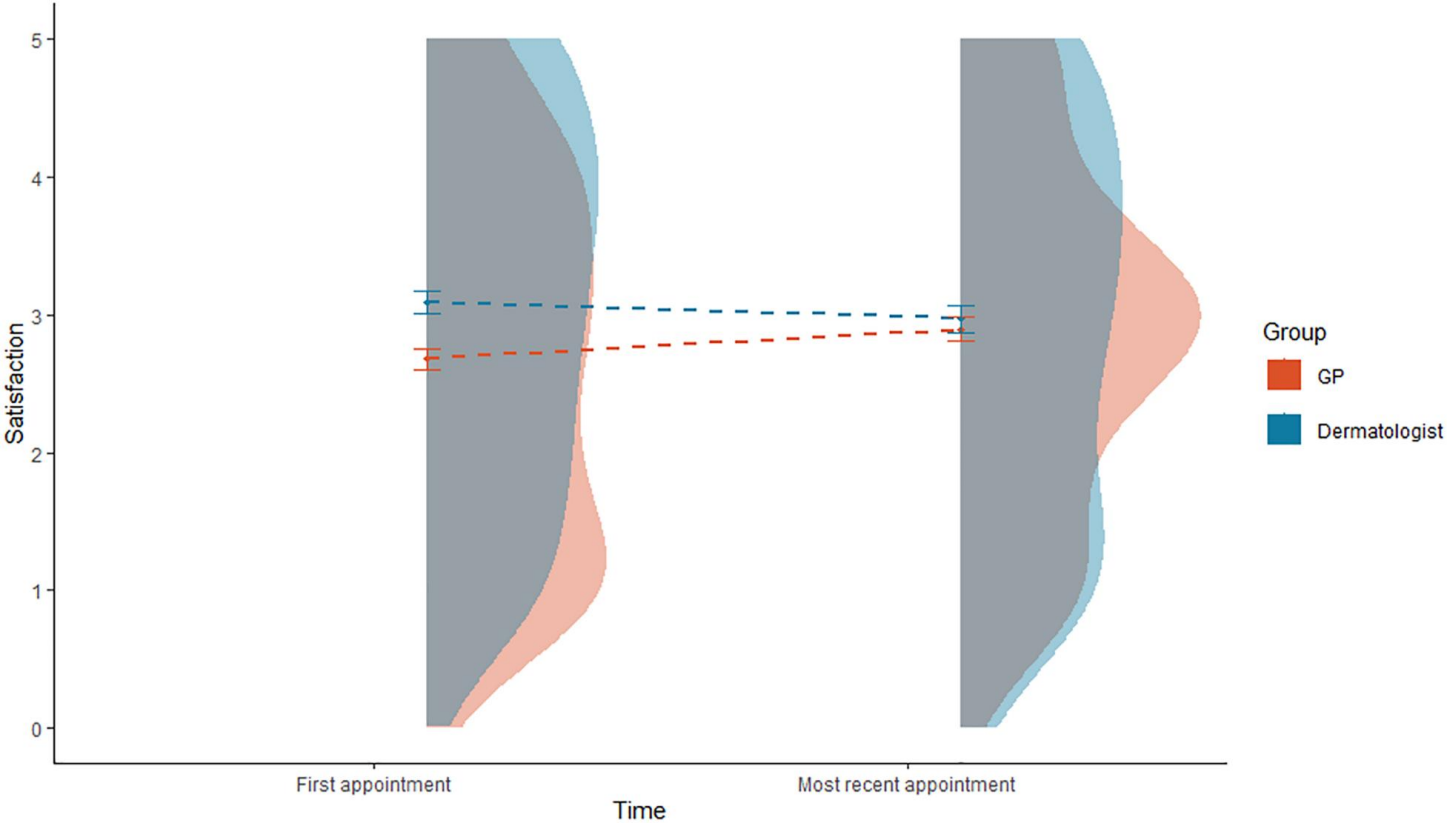
Raincloud plot comparing satisfaction with General Practitioner (GP) and dermatologist appointments using boxplots with interquartile ranges, violin plots and means over time.

A comparison of satisfaction rates between GP and Dermatologist appointments is shown in Figure 2. People were more satisfied with their first dermatologist appointment (*M* = 3.09) than their first GP appointment (*M* = 2.71; *t*(304) = 3.87, *p* < 0.001, *d* = 0.22), and there were no significant differences between the most recent dermatologist appointment (*M* = 2.94) and the most recent GP appointment (*M* = 2.96; *t*(182) = −0.144, *p* = 0.886, *d* = 0.01).

### 3.9 Suggested improvements for healthcare professionals

**FIGURE 3:**
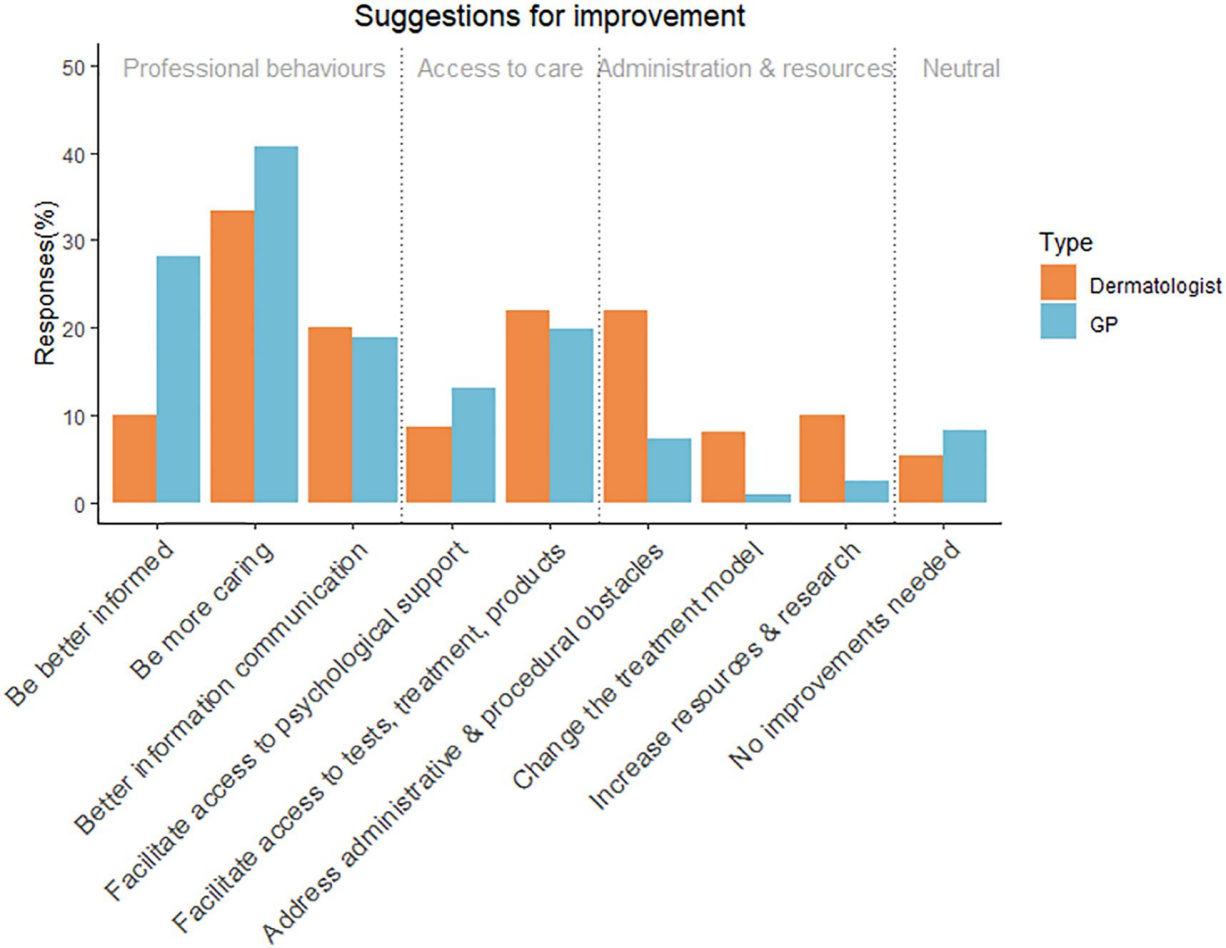
Content analysis of participants' suggestions for improvement to General Practitioner (GP) and dermatologist services.

Participants provided textual suggestions on how GP and dermatologist services could be improved. Using content analysis, codes were grouped into the four categories shown below. All codes are shown in Figure 3, and code descriptions are given in Supplementary Table 2. A total of 206 suggestions for improvements to GP services and 150 for dermatologist services were made.

### 3.10 Healthcare professional behaviours

The code with the greatest number of responses was “be more caring”, both for GP and dermatologist services. Examples of participants' relevant responses were:

Alopecia is such a traumatic journey. GPs need to deal with patients with sensitivity.

The Dermatologist should take mental health and general health into consideration, not just look any head [sic] and dismiss me as untreatable.

Approximately one‐fifth of respondents reported that their experiences would be improved by better communication of information by GPs, and the same proportion reported this regarding their Dermatology experiences. Example quotes include:

Could have been improved with a bit more information about alopecia as [the GP] didn't really give any information or answers in the time frame waiting for a dermatology [appointment], which is stressful.

More information [from the dermatologist] about alopecia and tests available and treatments would have been helpful.

Over a quarter of participants suggested that their GP experience could be improved by GPs being better informed about alopecia, its management and available support:

I felt I knew more than my GP about the condition. I had already accepted that I was “on my own” and had to make my own decisions about how to handle my alopecia.

### 3.11 Access to care

Around a fifth of participants highlighted a need for both GPs and Dermatologists to better facilitate access to tests, treatment, and products, for example:

GPs need to be quicker to refer to Dermatology.

I think I had already accepted that nothing could be done. I was just given the facts [by the dermatologist]‐ no mention was made of any possible treatments, or even the possible supply of a wig.

Around a tenth of participants also wanted GPs and dermatologists to facilitate greater access to psychological support:

For years I have had to cope with this alone. No support. I was 18 when I lost my hair. No health care professional during my life so far has ever suggested that there is help or support out there.

### 3.12 Administration, resources and the medical approach

Just over a fifth of participants felt that it was important to address administrative and procedural obstacles in Dermatology services:

Unfortunately been waiting 15 months for latest dermatology appointment, it has been cancelled 3 times already, I don’t hold out much hope of seeing them this year.

Some responses also highlighted participants' frustration with the care pathway between primary and secondary care in alopecia management:

Having a system where patients with AA do not have to keep going back through GPs for referrals to dermatology for wigs or further treatment when it comes back.

### 3.13 No improvements warranted

Under 10% of people thought there were no improvements warranted for GPs and dermatologists:

I’m not sure how it can be improved. GPs have to deal with such a wide range of issues they can’t possibly be experts in everything.

I have had nothing but positive experiences with the Dermatology department in my local hospital.

### 3.14 Satisfaction with psychological support

Overall, people rated their satisfaction with the psychological support for living with alopecia with a mean of 1.99, closest to the fairly dissatisfied rating. A small proportion were either fairly satisfied (*n* = 21, 5.82%) or very satisfied (*n* = 13, 3.60%). Neutral ratings were given by 47 people (13.02%). The majority were either fairly dissatisfied (*n* = 77, 21.33%) or very dissatisfied (*n* = 130, 36.01%), signalling that psychological support for those of alopecia can be improved in the NHS.

## 4 DISCUSSION

This study investigated patients' experiences of NHS care provision for alopecia in the UK for the first time. Overall, participants reported somewhere between mild dissatisfaction and neutral experiences with their GP consultations (2.60–2.89 on 1‐5 ratings) and neutral experiences with dermatologists (2.97–3.17). Drawing from broadly comparable data, these findings suggest individuals with alopecia are on average less satisfied than the UK general population with their health care experiences. That is, on 1‐5 ratings, in the current study GP satisfaction was 2.60–2.89, and dermatology satisfaction was 2.97–3.17; whereas the general population scored 4.12 on average for both GP and hospital outpatient consultations.^26^

An apparent deficit in GPs' knowledge of alopecia was also raised by participants as a common explanation of their dissatisfaction, with a third of the total sample reporting this issue. This may also help to explain the finding that participants were more satisfied with their first dermatologist appointment compared to their first GP appointment, given that GPs likely lack the specialist knowledge that dermatologists possess, and may be comparatively less experienced with patients presenting with alopecia. However, any beneficial effect of this knowledge and experience on patient satisfaction for dermatologists was not evident when participants reflected on their most recent appointments, with no difference between GP and dermatology satisfaction ratings. This may reflect the lack of effective hair restoration treatment and/or care received by patients from dermatologists, in the UK as well as internationally.^27^, ^28^ The relatively rare nature of alopecia may well account for GPs' apparent gap in knowledge, in contrast to general population findings in which 90% patients agreed or strongly agreed that their GP had been knowledgeable about their health condition.^26^ The finding that participants were more satisfied with their most recent compared to their first GP appointment could point to modified GP behaviour and/or reduced patient expectations over this period.

Overall, these findings indicate a need for greater GP training on alopecia. This may be especially pertinent given reports of overly lengthy delays for dermatology referrals, during which time participants sometimes reported feeling lost and alone. The minimal research published globally on the topic of GPs' alopecia knowledge indicates that such training would be warranted internationally. For example, in a survey of GPs in Saudi Arabia, over 15% of GPs thought that alopecia is a communicable disease and over a quarter blamed personal hygiene.^29^

Along with concerns about GPs' condition‐specific knowledge, participants also commonly reported receiving an unkind or uncaring response both from their GP and their dermatologist. Relatedly, being more caring was the most reported suggestion for improvement, and was especially common in reference to GPs. This emphasises the importance of receiving a caring and empathic response from health professionals when adjusting to an often unpredictable and poorly understood condition, which can have a profound effect on psychological well‐being.^5^, ^6^ This aligns with published research from the U.S. in which alopecia patients who reported low patient satisfaction were more likely to simultaneously report subclinical anxiety and depression.^28^

These findings suggest that future practice guidelines in the UK and beyond may benefit from highlighting the centrality of empathic communication with a patient group who often appear to feel their experience is poorly understood and minimised. Current guidelines are empirically grounded, yet from our findings it appears that the way the information is delivered (namely in a caring or non‐caring way) plays an important role alongside the actual content of the message when it comes to patient satisfaction. This was apparent in the “helpful information” and “unhelpful information” codes. Participants' accounts of being told that they may not experience hair regrowth again, for example, were placed in either the “helpful” or “unhelpful” category by virtue of how helpful the participant perceived this prognosis to be, which often appeared to be influenced by the style in which it was delivered. Given the relative dearth of available efficacious treatments for most forms of alopecia^27^ despite more promising treatments on the horizon,^30^ in practise there may be a difficult balance for healthcare practitioners to strike between offering hope and reassurance, and carefully managing patient expectation. Honest, empathic communication may help resolve this tension.

Participants' suggestions for improvements to GP and dermatology services also point to the need for a more joined up and simplified care pathway between primary care, secondary care, mental health services and third‐party providers, inferred from categories such as “facilitate access to tests, treatments & products”, “facilitate access to psychological support” and “address administrative & procedural obstacles”. This principle is also applicable internationally, including in insurance‐based health care systems, to increase cost effectiveness for the affected individuals, health providers, tax‐payers and insurance companies. Indeed, recent research from the U.S. shows alopecia incurs greater healthcare costs compared to matched controls through surgical procedures, pharmacological and psychological interventions,^31^ suggesting scope for greater efficiency in the care pathway.

On average, patients were fairly dissatisfied with the psychological support available for people with alopecia and about a tenth of participants indicated that access to psychological support should be facilitated by GPs and dermatologists. Together, these findings point to a greater need for accessible and appropriate psychological support.

Future studies should consider using in‐depth interviews to assess how people with alopecia experience the entire care pathway both in the UK and internationally. Research should also focus specifically on understanding the often‐underrepresented experiences of those from Black and Asian minority ethnic groups, who are overrepresented in alopecia prevalence in the UK.^11^ Follow‐up to the indicative findings regarding dissatisfaction with psychological support is also warranted.

## 5 STRENGTHS AND LIMITATIONS

A strength of this study is its large sample from multiple regions across the UK. Our sample thus represents multiple NHS service providers and opinions across regions.

A first limitation is that no demographic data were available for age, gender, ethnicity/race, or socioeconomic status, which precluded analysis of satisfaction ratings by demographic data. This is particularly unfortunate in light of recent findings suggesting higher rates of AA in Asian ethnicity groups and those living in urban and socially deprived areas in the UK.^11^ We could at least make an informed estimate that the sample was overwhelmingly female, with an average age of 37–42. With males making fewer visits to their GP for alopecia and being less likely to be referred for dermatology in the UK,^11^ the experience of men with alopecia remains largely unexplored.

Secondly, the current data cover only limited snapshots of the patient journey via people's experiences of their first and most recent NHS appointments. Therefore, we were unable to draw conclusions on patients' overall journeys. More in‐depth qualitative methods like interviews may allow greater exploration of the entire experience. A last limitation is that the survey contained only one question on psychological support, and as a result could not illuminate the reasons for participants' general dissatisfaction with available provision.

## 6 CONCLUSIONS

These limitations notwithstanding, this study provides novel insight on the experience of going through primary and specialist health care as a patient with alopecia. To help health care providers manage patients' expectations and optimise overall patient experience, it appears important to increase GPs' medical knowledge on alopecia, and for all health professionals including Dermatologists to understand the potential psychosocial impact (positive or negative) of their consultations, and to employ empathic communication when consulting affected individuals.

## CONFLICT OF INTEREST STATEMENT

The authors have no conflicts of interests to disclose.

## AUTHOR CONTRIBUTIONS

**Fabio Zucchelli**: Formal analysis (equal); Methodology (equal); Project administration (equal); Writing – original draft (equal); Writing – review & editing (equal). **Marije van Dalen**: Software (lead); Visualisation (equal); Writing – original draft (equal); Writing – review & editing (equal). **Nick Sharratt**: Formal analysis (equal); Methodology (equal); Project administration (equal); Writing – review & editing (supporting). **Amy Johnson**: Conceptualisation (equal); Data curation (lead); Investigation (equal); Methodology (equal); Project administration (equal); Writing – review & editing (supporting). **Jen Chambers**: Conceptualisation (equal); Data curation (supporting); Funding acquisition (equal); Investigation (equal); Methodology (equal); Project administration (equal); Writing – review & editing (supporting).

## ETHICS STATEMENT

Retrospective ethical approval for this study was granted by the University of the West of England Health and Applied Sciences Faculty Ethics Committee, reference number HAS.20.04.164. The authors assert that all procedures contributing to this work comply with the ethical standards of the relevant national and institutional guidelines on human experimentation and with the Helsinki Declaration of 1975, as revised in 2008.

## Supporting information

Supplementary Material

## Data Availability

The data that support the findings of this study are available on request from the corresponding author. The data are not publicly available due to privacy or ethical restrictions.
