# Supplementary material for: Patients' experiences of primary healthcare and dermatology provision for alopecia
Source: Skin Health Dis. 2023 Dec 10;4(2):e324. doi: 10.1002/ski2.324 (PMC10988748; doi:10.1002/ski2.324)
Supplement: Supplementary file 1 — Supplementary Material [file SKI2-4-e324-s001.docx]

| *Category* and Codes | Code description |
| --- | --- |
| *Positive experiences* |  |
| Helpful information | Provided with helpful, useful, or accurate information or explanation about alopecia; including information connected to diagnosis, treatment, and/or prognosis. |
| Caring response | Response was experienced as understanding, kind, caring, compassionate, sensitive, empathetic, reassuring, supportive, sympathetic, professional, interested, attentive, and/or as facilitating positive feelings or experience. |
| Facilitated testing, treatment and/or access to care  *(GP only)* | GP organised or facilitated access to onward referral, tests, treatment, and/or products (including wigs); Outlined care pathway. |
| Positive investigation &  treatment experience *(dermatologist only)* | Thorough and/or satisfactory testing, treatment (including unspecified treatment, injections, creams and/or chemical treatments) and/or products (including wig provision). |
| *Negative experiences* | |
| Unhelpful and/or inadequate information | HP was felt to lack knowledge and/or not provide any helpful, accurate or appropriate information, advice, or explanation about alopecia, its diagnosis, treatment, and/or prognosis. This included cases of misdiagnosis. |
| Uncaring and/or unkind response | HP’s personal response was experienced as unsympathetic, lacking, compassion or concern, unsupportive, disinterested, insensitive, not reassuring. This included a sense of abandonment, HP being actively unkind, dismissive, disinterested, flippant, passive or inactive, and failing to acknowledge the emotional/psychological impact upon the respondent. |
| Obstructed testing, treatment and/or access to care *(GP only)* | A failure or delay in offering referral to other services to provide access to further testing, products (including wigs), psychological support, treatment, prescriptions. This included failing to provide information about the process of referral. |
| Administrative obstructions to care *(dermatologist only)* | Delayed or long waits for appointments, administrative issues (including missing notes, appointment letters not being sent), a lack of information-sharing between departments. |
| Negative experience of treatment or products | Being offered or advised alopecia treatment or products (including wigs) that were not effective or were otherwise unsatisfactory, being offered or prescribed other, unconnected medication (such as antidepressants). |
| Could or would not help | Being told that there is nothing that can be done, the HP cannot help, there is no cure or treatment, that there are no new treatment options. The respondent reporting having to or actually utilising or being told to utilise private care (treatment, products), do their own research, and/or educate and advise their HP about alopecia. |
| *Neutral experiences* |  |
| Neutral information, investigation and/or treatment experience | Descriptive only statements that, when the language used and preceding ratings were considered, suggested neutral feelings on the topic. This included the lack of ongoing HP involvement and the advice to watch and wait where this was evaluated neutrally as well as some instances of respondents being unable to remember  their consultations. |

Table S1. Code descriptions for participants’ experiences of GP and Dermatology care provision

Table S2. Code descriptions for participants’ suggestions for improvement to GP and dermatologist services

| *Category* and code name | Code description |
| --- | --- |
| *Healthcare professional behaviours* | |
| Be better informed | The relevant HP should be possess greater knowledge and/or awareness of alopecia, its treatment, and/or services. |
| Better Information Communication (regarding alopecia, diagnosis, prognosis) | The relevant HP should communicate a greater quantity or better quality of information, or provide information or advice, and/or respond to questions relating to alopecia and its diagnosis, prognosis, testing, treatments, choices and options. |
| Be more caring | The relevant HP should be more empathic, more caring, take the patient and/or their condition seriously, listen to the patient. They should give sufficient time to the patient and ensure patient privacy is respected. |
| *Access to care* |  |
| Facilitate access to tests, treatment, and products | The relevant HP should facilitate or refer patients on for the performance of appropriate and/or extensive diagnostic tests, checks, or examinations (in a timely fashion), provide and/or prescribe appropriate or thorough treatment and/or services (including wigs). |
| Facilitate access to psychological support | The relevant healthcare professional(s) should signpost or link to sources of emotional and psychological support for patients, offer or refer to counselling services, and/or focus more intently on the emotional and psychological impact of alopecia. |
| *Administration, resources and the medical approach* | |
| Address administrative & procedural obstacles | Administrative and procedural issues and inconveniences should be rectified, including dermatologist waiting times, unnecessary movement between GP and dermatologist care, , continuity of healthcare personnel, and appointment cancellations and delays. |
| Increase resources and research | More research, resources, treatments, specialists, and/or clinics are required. |
| Change the treatment model | The current model for treating alopecia should be altered to a more holistic approach focussing on cause rather than symptoms, and/or there should be healthcare professionals who specialise in hair-loss. |
| *No improvements warranted* | |
| No improvements needed | No improvements are warranted, required, and/or justified, nothing more could be done. |
